# Obstetric blood transfusion in placenta previa patients with prenatal anemia: a retrospective study

**DOI:** 10.1186/s12884-024-06279-4

**Published:** 2024-01-30

**Authors:** Baolian Zhang, Hong Liu, Haiyan Li, Jia Wang, He Zhu, Peijia Yu, Xianghua Huang, Wenli Wang

**Affiliations:** 1 Department of Physical Examination Center, The Second Hospital of Hebei Medical University, Shijiazhuang, China; 2 Department of Ultrasound in Obstetrics and Gynecology, The Second Hospital of Hebei Medical University, Shijiazhuang, China; 3 Department of Quality Control, The Second Hospital of Hebei Medical University, Shijiazhuang, China; 4 Department of Gynecology and Obstetrics, The Second Hospital of Hebei Medical University, 215 West Heping Road, Shijiazhuang, 050000 China; 5 Department of Medical Record, The Second Hospital of Hebei Medical University, Shijiazhuang, China

**Keywords:** Placenta previa, Prenatal anemia, Obstetric blood transfusion, Hemoglobin, Retrospective

## Abstract

**Background:**

The appropriate use of obstetric blood transfusion is crucial for patients with placenta previa and prenatal anemia. This retrospective study aims to explore the correlation between prenatal anemia and blood transfusion-related parameters in this population.

**Methods:**

We retrieved the medical records of consecutive participants who were diagnosed with placenta previa and underwent cesarean section in our hospital. We compared the baseline demographics and clinical characteristics of patients with and without anemia. The correlation between prenatal anemia and obstetric blood transfusion-related parameters was evaluated using multivariate regression analysis.

**Results:**

A total of 749 patients were enrolled, with a mean prenatal hemoglobin level of 10.87 ± 1.37 g/dL. Among them, 54.87% (391/749) were diagnosed with anemia. The rate of obstetric blood transfusion was significantly higher in the anemia group (79.54%) compared to the normal group (44.41%). The median allogeneic red blood cell transfusion volume in the anemia group was 4.00 U (IQR 2.00–6.00), while in the normal group, it was 0.00 U (IQR 0.00–4.00). The prenatal hemoglobin levels had a non-linear relationship with intraoperative allogeneic blood transfusion rate, massive blood transfusion rate, red blood cell transfusion units, and fresh plasma transfusion volume in patients with placenta previa, with a threshold of 12 g/dL.

**Conclusions:**

Our findings suggest that prenatal anemia is associated with a higher rate of blood transfusion-related parameters in women with placenta previa when the hemoglobin level is < 12 g/dL. These results highlight the importance of promoting prenatal care in placenta previa patients with a high requirement for blood transfusion.

## Introduction

Placenta previa, a condition in which the placenta partially or completely covers the internal os of the cervix after the 28th week of pregnancy, is associated with a rising prevalence. This increase is positively correlated with advanced maternal age and a history of previous cesarean section [1–3], making it crucial to address this condition promptly. Pregnant women diagnosed with placenta previa require emergent medical care before, during, or after delivery due to the potential for danger or life-threatening complications. This condition not only impedes safe vaginal delivery but also mandates the delivery of newborns by cesarean section.

Patients with placenta previa face a heightened risk of anemia, a common pregnancy complication known to lead to adverse maternal–fetal outcomes [4–6]. Hemoglobin, a crucial component of red blood cells responsible for oxygen transport, is integral to maintaining adequate oxygenation for both the mother and the developing fetus. Antepartum anemia can elevate nitric oxide production, enhancing its biologic effects, which may result in uterine muscle relaxation and contribute to atonic perioperative and postpartum hemorrhage [7–13]. Several studies have explored the intricate interplay between anemia and the pregnancy outcomes, both maternal and fetal. Shi et al. found that the severity of anemia during pregnancy correlated with an elevated risk of adverse events, including placental abruption, preterm birth, severe postpartum hemorrhage, and fetal malformations. [14] Another population-based study demonstrated that anemia during pregnancy was associated with increased risks of peri-partum, intra-partum, and post-partum complications for the mother, as well as a higher likelihood of preterm birth for the infant [6]. A longitudinal study involving 2000 pregnant women revealed significant differences in prematurity, birth weight, and hypertensive disorders of pregnancy between anemic and nonanemic groups, emphasizing the broad-ranging impact of anemia on pregnancy outcomes [15]. Therefore, raising awareness of anemia is essential for tailoring effective management strategies to optimize maternal and fetal well-being in pregnant women with placenta previa.

Obstetric blood transfusion has emerged as a life-saving strategy for managing hemorrhage caused by anemia. In cases of acute bleeding, the role of blood transfusion is to maintain tissue oxygenation and reverse or prevent coagulation. Obstetric blood transfusion involves the use of allogeneic blood products during and up to 24 h after delivery. When the hemoglobin level falls below 7 g/dL, blood transfusion is often necessary to reduce the prevalence rate of bleeding and mortality [16]. Proper use of blood component transfusion is crucial for meeting transfusion needs and preventing transfusion reaction risks such as allergy, acute immune hemolysis, or delayed hemolysis.

This retrospective study aims to evaluate the association of prenatal anemia with obstetric blood transfusion-related parameters in patients diagnosed with placenta previa.

## Methods

### Participants and study design

This is a retrospectively study, and we retrospectively reviewed the medical records of pregnant women with placenta previa who gave birth at the Department of Obstetrics of the Second Hospital of Hebei Medical University between January 2014 and December 2020 [17]. Eligible patients underwent cesarean section and had a gestational age of 28–42 weeks. Those with hematological disorders, coagulopathy, thrombocytopenia, or missing data on prenatal anemia and obstetric blood transfusion were excluded.

The requirement for informed consent was waived due to the retrospective and anonymous nature of the data. The study received approval from the Ethics Committee of the Second Hospital of Hebei Medical University (approval No. 2019-R085).

### Procedures

Patient information was retrieved from electronic medical records using International Classification of Diseases 10th edition (ICD O44) codes. Demographic and clinical data were collected using EpiDatav3.1 software, covering baseline characteristics such as age, occupation, region, body mass index, pregnancy complications, gravidity, parity, number of previous cesarean sections, surgical history, placental details, and hemorrhage during pregnancy.

### Assessments

Maternal hemoglobin concentration was determined preoperatively using a blood cell analyzer (UniCel DxH 800, Beckman Coulter). Prenatal anemia was defined as hemoglobin concentration < 11.0 g/dL according to the World Health Organization (WHO) guidelines [18]. Massive blood transfusion was defined as transfusion of ≥ 10 units of allogeneic red blood cells. Placental location, placenta previa types, placenta accreta spectrum, and fetal presentation were examined preoperatively using the American GE-E10 color Doppler ultrasound diagnostic instrument. Examination involved transabdominal or transabdominal combined with transvaginal scanning [19].

### Statistical analysis

No formal statistical assumptions of samples size were involved in this study. Continuous variables with normal distribution were presented as mean ± standard deviation (SD), and between-group differences were compared using analysis of variance (ANOVA). Continuous variables with skewed distribution were presented as median (interquartile range [IQR]), and the Kruskal–Wallis rank-sum test was used to compare the differences between groups. Categorical variables were presented as number (percentage), and inter-group comparisons were performed using the Chi-Square test. If the theoretical count for a categorical variable is < 10, *P* values were obtained using Fisher's exact probability test.

Multivariable regression analyses were conducted to explore the correlation between anemia in patients with anterior placenta previa and intraoperative blood product use. A generalized estimating equation model (GEE) was used to explore the association between prenatal anemia and intraoperative allogeneic blood transfusion, as recommended in the STROBE statement [20]. Three models were used to adjust for potential confounders. Model A was the non-adjusted model. Age (continuous), region, occupation, body mass index (continuous), placenta previa types, placental location, and placenta accreta spectrum were adjusted for in model B, as described in previous studies [21–23]. Region, occupation, number of cesarean sections, uterine anomalies (bicornuate uterus, adenomyosis, uterine fibroids, or prior uterine surgery), hemorrhage during pregnancy, emergency surgery, placental location, placenta previa types, and placenta accreta spectrum were adjusted for in model C based on their association with the outcomes of interest or a change in effect estimate above 10%. Multiple imputation was used to evaluate the sensitivity of missing data. The results of group comparisons were presented as odds ratios (ORs) with a 95% confidence interval (CI). Additionally, a generalized additive model with a spline smoothing function was used to explore the non-linear relationship between prenatal anemia level and intraoperative blood transfusion.

All analyses were performed using R statistical software (http://www.R-project.org, The R Foundation) and Empower Stats (http://www.empowerstats.com, X&Y Solutions, Inc., Boston, MA). Statistical significance was set at *P* < 0.05 and all analyses were two-tailed.

## Results

### Baseline demographics and clinical characteristics of participants

This study analyzed a total of 749 participants diagnosed with placenta previa. The baseline demographics and clinical characteristics of the study population are presented in Table 1.

**Table 1:** Baseline demographics and clinical characteristics

Characteristics	Total (*n* = 749)	Anemia (*n* = 391)	Normal (*n* = 358)	*P* value
Maternal age, mean ± SD, years	31.36 ± 4.64	31.02 ± 4.72	31.72 ± 4.53	0.041
Region^a^				< 0.001
Rural	495	281 (56.77%)	214 (43.23%)	
Urban	253	110 (43.48%)	143 (56.52%)	
Unknown	1	0	1 (0.28%)	
Occupation				0.019
No	513 (68.49%)	283 (72.38%)	230 (64.25%)	
Yes	235 (31.38%)	108 (27.62%)	127 (35.47%)	
Unknown	1 (0.13%)	0	1 (0.28%)	
Body mass index, kg/m^2^	28.49 ± 3.92	28.23 ± 4.05	28.76 ± 3.76	0.067
Pregnancy complications	217 (28.97%)	103 (26.34%)	114 (31.84%)	0.097
Gestational age at delivery, weeks	35.33 ± 2.30	35.06 ± 2.41	35.62 ± 2.14	0.806
Gravidity				0.023
2	203 (27.10%)	93 (23.79%)	110 (30.73%)	
3	193 (25.77%)	115 (29.41%)	78 (21.79%)	
≥ 4	353 (47.13%)	183 (46.80%)	170 (47.49%)	
Parity				0.051
0	75 (10.01%)	31 (7.93%)	44 (12.29%)	
1	480 (64.09%)	245 (62.66%)	235 (65.64%)	
2	166 (22.16%)	99 (25.32%)	67 (18.72%)	
≥ 3	28 (3.74%)	16 (4.09%)	12 (3.35%)	
Previous caesarean section	600 (80.11%)	327 (83.63%)	273 (76.26%)	0.012
Previous vaginal delivery	114 (15.22%)	55 (14.07%)	59 (16.48%)	0.358
Uterine anomalies	479 (63.95%)	258 (65.98%)	221 (61.73%)	0.226
Assisted reproduction technology	34 (4.54%)	18 (4.60%)	16 (4.47%)	0.930
Emergency surgery	179 (23.90%)	100 (25.58%)	79 (22.07%)	0.261
Hemorrhage during pregnancy	468 (62.48%)	250 (63.94%)	218 (60.89%)	0.390
Multiple pregnancy	21 (2.80%)	15 (3.84%)	6 (1.68%)	0.074
Placental location				0.012
Posterior	166 (22.16%)	74 (9.88%)	92 (25.70%)	
Lateral	49 (6.54%)	19 (2.54%)	30 (8.38%)	
Anterior	131 (17.49%)	69 (17.65%)	62 (17.32%)	
Central	399 (53.27%)	227 (58.06%)	172 (48.04%)	
Unknown	4 (0.53%)	2 (0.51%)	2 (0.26%)	
Placenta previa types				0.013
Low-lying	10 (1.34%)	4 (1.02%)	6 (1.68%)	
Marginal	111 (14.82%)	43 (11.00%)	68 (18.99%)	
Partial	61 (8.14%)	36 (9.21%)	25 (6.98%)	
Central	567 (75.70%)	308 (78.77%)	259 (72.35%)	
Placenta accrete spectrum	693 (93.02%)	366 (94.09%)	327 (91.85%)	0.232
Fetal presentation				0.630
Cephalic	599 (80.08%)	318 (81.33%)	281 (78.71%)	
Breech	98 (13.10%)	47 (12.02%)	51 (14.29%)	
Shoulder	51 (6.82%)	26 (6.65%)	25 (7.00%)	
Unknown	1 (0.13%)	0	1 (0.28%)	
Prenatal hemoglobin, g/dL	10.87 ± 1.37	9.82 ± 0.85	12.01 ± 0.82	< 0.001

Data are presented as mean ± SD or n (%)

^a^The percentages were calculated using the total numbers 495 and 253 as the denominators for rural and urban, respectively

Among the 749 enrolled patients, 52.20% (391/749; 95% CI 50.91–58.86) of the total patients were diagnosed with prenatal anemia. The occurrence of prenatal anemia showed a gradual decline over the study period (2014–2020, Fig. 1). The prenatal hemoglobin level of the total patients was 10.87 ± 1.37 g/dL (mean ± SD), with 9.82 ± 0.85 g/dL in the anemia group and 12.01 ± 0.82 g/dL in the normal group (*P* < 0.001).

**Fig. 1:**
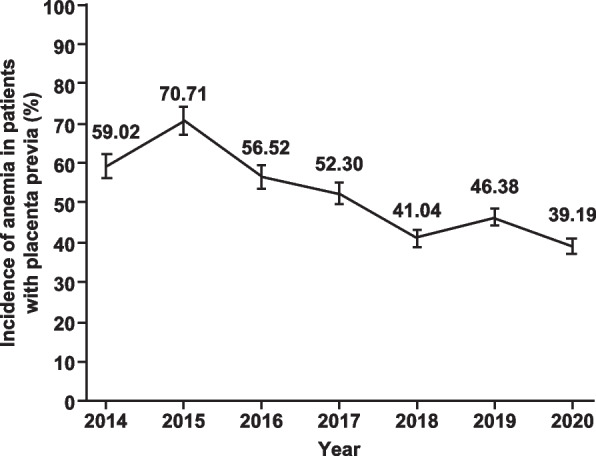
The prevalence of anemia in patients with placenta previa over the study period from 2014 to 2020

Compared with the normal group, the prenatal anemia group showed lower values of maternal age (31.02 ± 4.72 vs 31.72 ± 4.53 years, mean ± SD), were more likely to be from rural areas (56.77% vs 43.23), had no occupation (72.38% vs 64.25%), higher gravidity (≥ 3, 76.21% vs 69.27%), were more likely to have a history of caesarean Sect. (83.63% vs 76.26%), exhibited placental central location, and were more likely to have partial placenta previa (all *P* < 0.05).

### Comparison of obstetric blood transfusion-related parameters in the prenatal anemia group and the normal group

The median volume of intraoperative blood loss (1500 mL vs. 1050 mL) in the anemia group was significantly higher than that of the normal group (*P* < 0.05). With respect to obstetric blood transfusion-related parameters, placenta previa patients with prenatal anemia had higher rates of obstetric blood transfusion (79.54% vs. 44.41%), massive blood transfusion (12.53% vs. 1.68%), and fresh plasma transfusion (75.45% vs. 43.85%), more units of intraoperative red blood cell transfusion (4.00 U vs. 0 U), and more volume of fresh plasma transfusion (400.00 mL vs. 0 mL), compared with placenta previa patients without prenatal anemia (all *P* < 0.05; Table 2).

**Table 2:** Obstetric blood transfusion-related parameters between groups

Characteristics	Total (*n* = 749)	Anemia (*n* = 391)	Normal (*n* = 358)	*P* value
Blood loss, mL	1200.00 (800.00–2200.00)	1500.00 (805.00–2600.00)	1050.00 (605.00–1600.00)	< 0.001
Obstetric blood transfusion rate	470 (62.75%)	311 (79.54%)	159 (44.41%)	< 0.001
Massive blood transfusion rate ^a^	55 (7.34%)	49 (12.53%)	6 (1.68%)	< 0.001
Red blood cell transfusion, U	2.00 (0.00–4.00)	4.00 (2.00–6.00)	0.00 (0.00–4.00)	< 0.001
Fresh plasma transfusion rate	452 (60.3%)	295 (75.45%)	157 (43.85%)	< 0.001
Fresh plasma transfusion, mL	400.00 (0.00–450.00)	400.00 (150.00–600.00)	0.00 (0.00–400.00)	< 0.001
Platelets transfused, U	0.00 (0.00–0.00)	0.00 (0.00–0.00)	0.00 (0.00–0.00)	NA

Data are presented as median (IQR) or n (%)

^a^Massive blood transfusion is defined as the transfusion of 10 units of packed red blood cells within a 24-h period

### Association of prenatal anemia with specific obstetric blood transfusion-related parameters

Multivariate regression models were used to explore the association of prenatal anemia with the above five obstetric blood transfusion-related parameters that were significantly different between the two study groups (Table 3). The adjusted models (model b and model c) showed a significant correlation between prenatal anemia with blood transfusion rate, massive blood transfusion rate, intraoperative allogeneic red blood cell units, and fresh plasma transfused volume. Unadjusted model (model a) indicated that the obstetric blood transfusion rate (OR 4.87, 95% CI 3.53–6.72) and massive blood transfusion rate (8.41, 95% CI 3.55–19.87) were significantly positively correlated with anemia. The values of non-adjusted ORs were similar to that of the adjusted ORs.

**Table 3:** Association between maternal anemia and maternal outcomes in patients with placenta previa

Outcomes	Normal	Anemia		
		**OR/β (95% CI)**^**a**^	**aOR/β (95% CI)**^**b**^	**aOR/β (95% CI)**^**c**^
Blood transfusion rate	1.0	4.87 (3.53, 6.72)	4.98 (3.48, 7.13)	4.89 (3.39, 7.06)
Red blood cell transfusion units	0	2.72 (2.21, 3.23)	2.45 (1.94, 2.96)	2.29 (1.80, 2.77)
Massive blood transfusion rate	1.0	8.41 (3.55, 19.87)	6.90 (2.88, 16.51)	7.33(3.01, 17.81)
Fresh plasma transfusion rate	1.0	3.93 (2.88, 5.37)	3.88 (2.73, 5.51)	3.91 (2.72, 5.62)
Fresh plasma transfusion volume	0	216.25 (166.09, 266.42)	181.76 (132.72, 230.79)	167.07 (120.98, 213.16)

Multivariable regression analyses were conducted to explore the correlation between anemia in patients with anterior placenta previa and intraoperative blood product use

*Abbreviations*: *OR* odds ratio, *CI* confidence interval

^a^Non-adjusted model

^b^Adjusted for age (continuous), region, occupation, BMI (continuous), type of placenta previa, placental location, placenta accrete spectrum

^c^Adjusted for region, occupation, Number of caesarean sections, uterine anomalies (bicornuate uterus, adenomyosis, uterine fibroids or prior uterine surgery), hemorrhage during pregnancy, emergency surgery, placental location, placenta previa types, and placenta accrete spectrum. Massive blood transfusion is defined as the transfusion of 10 units of packed red blood cells within a 24-h period

### Non-linear relationship of prenatal hemoglobin level with specific obstetric blood transfusion-related parameters

**Fig. 2:**
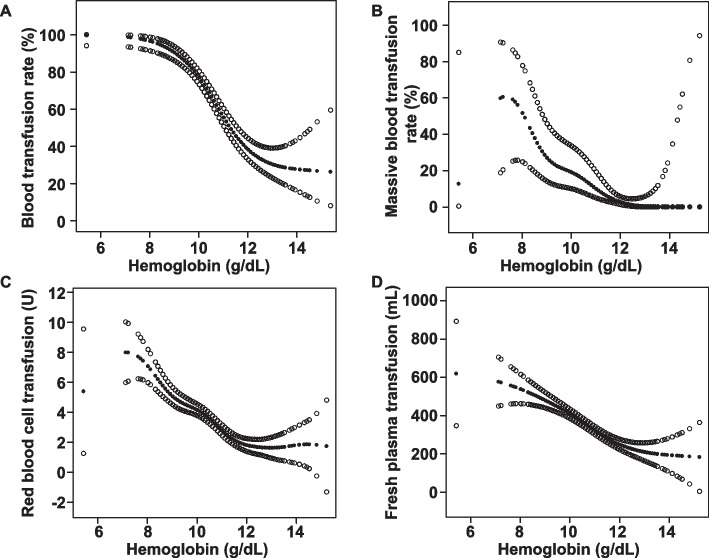
Correlation between prenatal hemoglobin levels and intraoperative blood transfusion-related parameters **A** Blood transfusion rate. **B** Massive blood transfusion rate. **C** Red blood cell transfusion units. **D** Fresh plasma transfused volume. The generalized additive model with a spline smoothing function (penalized spline method) was conducted to explore the nonlinear relationship between the prenatal hemoglobin level and intraoperative blood transfusion. The fitting curve is represented by solid dots, while the upper and lower 95% confidence intervals are represented by imaginary dots

A generalized additive model and smooth curve fitting were employed to investigate the relationship between prenatal anemia and the above four obstetric blood transfusion-related parameters that exhibited significant differences between the two study groups (Fig. 2). Adjusting for region, occupation, number of caesarean sections, uterine anomalies, hemorrhage during pregnancy, emergency surgery, placental location, placenta previa types, and placenta accrete spectrum, the prenatal hemoglobin level exhibited a non-linear relationship with obstetric blood transfusion rate, massive blood transfusion rate, intraoperative allogeneic red blood cell units, and fresh plasma transfused volume.

Furthermore, a two-piecewise linear regression analysis was conducted to identify the threshold effect of these parameters. The analysis indicated that when the prenatal hemoglobin level was below 12 g/dL, prenatal hemoglobin level was significantly negatively correlated with obstetric blood transfusion rate (OR 0.37, 95% CI 0.30–0.46), massive blood transfusion rate (OR 0.57, 95% CI 0.43–0.75), intraoperative allogeneic red blood cell units (aβ -1.18, 95% CI -1.41 to -0.95), and fresh plasma transfused volume (aβ -81.46, 95% CI -103.49 to -59.43). However, when the prenatal hemoglobin was greater than or equal to 12 g/dL, no significant association was observed between prenatal hemoglobin and obstetric blood transfusion rate, massive blood transfusion rate, intraoperative allogeneic red blood cell transfused units, or fresh plasma transfused volume (Table 4).

**Table 4:** Association between the level of maternal preoperative hemoglobin and maternal outcomes based on two-piecewise linear regression analysis

	Effect size adjusted β/OR	95% CI	*P*	*P* for the log-likelihood ratio test
Blood transfusion rate				< 0.001
Hemoglobin < 12 g/dL	0.37	(0.30, 0.46)	< 0.0001	
Hemoglobin ≥ 12 g/dL	1.03	(0.66, 1.60)	0.9093	
Massive blood transfusion rate^a^				0.033
Hemoglobin < 12 g/dL	0.57	(0.43, 0.75)	< 0.0001	
Hemoglobin ≥ 12 g/dL	0.00	(0.00, Inf)	0.9847	
Red blood cell transfusion units				< 0.001
Hemoglobin < 12 g/dL	-1.18	(-1.41, -0.95)	< 0.0001	
Hemoglobin ≥ 12 g/dL	0.04	(-0.56, 0.63)	0.9062	
Fresh plasma transfusion volume				0.039
Hemoglobin < 12 g/dL	-81.46	(-103.49, -59.43)	< 0.0001	
Hemoglobin ≥ 12 g/dL	-9.33	(-66.61, 47.96)	0.7498	

A two-piecewise linear regression model was used to examine the threshold effect of the prenatal hemoglobin level on intraoperative blood transfusion using a smoothing function. Likelihood ratio tests were conducted to compare the one-line linear regression model with a two-piecewise linear model

Adjusted for region, occupation, number of caesarean sections, uterine anomalies (bicornuate uterus, adenomyosis, uterine fibroids or prior uterine surgery), hemorrhage during pregnancy, emergency surgery, placental location, placenta previa types, and placenta accrete spectrum

Abbreviations: *OR* odds ratio, *CI* confidence interval

^a^Massive blood transfusion is defined as the transfusion of 10 units of packed red blood cells within a 24-h period

## Discussion

In our study, the prevalence of prenatal anemia in placenta previa patients was found to be 52.20%, which was higher compared with the prevalence reported in previous studies [24–26]. The high prevalence could be attributed to the high risk of postpartum bleeding during pregnancy [27], different levels of economic development, residence in the rural area [24], and lower educational level among the subjects. Our findings also showed that the prevalence of prenatal anemia in patients with placenta previa decreased from 59.02% in 2014 to 39.19% in 2020, which was consistent with previous studies indicating a downward trend in the prevalence of anemia in China from 2000 to 2019. The results demonstrated that prenatal hemoglobin levels were significantly correlated with the rate of intraoperative allogeneic blood transfusion, rate of massive blood transfusion, intraoperative allogeneic red blood cell units, and fresh plasma transfused volume in patients with placenta previa and a prenatal hemoglobin level of < 12 g/dL. Future studies are warranted to confirm our findings and investigate the role of different interventions in improving pregnancy outcomes in this patient population.

Clinical risk factors such as type of placenta previa, antepartum bleeding, placenta accrete spectrum, and history of C-section are associated with adverse pregnancy outcomes in placenta previa patients [12, 23, 28, 29]. Our findings also indicated that prenatal anemia was an independent risk factor for intraoperative allogeneic blood transfusion, massive blood transfusion, intraoperative allogeneic red blood cell transfusion units, and fresh plasma transfusion volume in placenta previa patients, which is consistent with previous studies [5, 27, 30]. Placenta previa patients with prenatal anemia were associated with high volumes of intraoperative blood loss compared with patients without anemia, which may be due to their lower reserves of red blood cells and increased risk for blood requirement, as shown by our findings and previous studies [31]. The smooth curve fitting and two-piecewise model indicated a significant association between hemoglobin level and the risk of intraoperative allogeneic blood transfusion, massive transfusion, intraoperative allogeneic red blood cell volume, and fresh plasma transfused volume, after adjusting for confounders in placenta previa patients. This finding implies that timely treatment of prenatal anemia is crucial for patients with placenta previa, and prospective multi-center studies should be conducted to further explore the causal relationship between prenatal hemoglobin level and pregnancy outcomes.

The present study had some limitations that need to be acknowledged. Firstly, it was a single-center retrospective analysis, which may have led to bias and one-sidedness of the findings. Secondly, several uterotonic agents, including tranexamic acid, and surgical approaches such as bilateral uterine artery ligation, B-lynch sutures, focal sutures, and cervical pull sutures were not used for adjustment factor, which might have underestimated the association between prenatal hemoglobin levels and intraoperative allogeneic red blood cell transfusion. Nonetheless, target independent variables were used as continuous variables, thereby enhancing the robustness of our results.

Our study findings have profound implications for the clinical management of placenta previa patients. Specifically, for pregnant women facing the dual challenge of placenta previa and anemia, there is a compelling emphasis on the necessity of early blood transfusion during pregnancy. This proactive measure aims to mitigate risks associated with antenatal anemia, ultimately enhancing overall pregnancy outcomes. Additionally, our results underscore the need for healthcare centers to adopt a proactive approach by establishing dedicated and robust blood transfusion backup for patients navigating the complexities of placenta previa and anemia. Furthermore, we emphasize the importance of promoting antenatal awareness, empowering patients to recognize and address anemia and placenta previa complications promptly. This increased awareness facilitates early intervention, allowing healthcare providers to manage these conditions more effectively during delivery, thereby lowering the risk of postpartum hemorrhage. The proposed prevention measures, including routine iron supplementation, early anemia screenings, and educational programs, align with successful interventions in managing pregnancy-related anemia, offering a feasible strategy to reduce the prevalence of prenatal anemia and associated complications in placenta previa patients.

## Data Availability

The data that support the findings of this study are available from the corresponding author upon reasonable request.
